# Adapting the Stress Response: Viral Subversion of the mTOR Signaling Pathway

**DOI:** 10.3390/v8060152

**Published:** 2016-05-24

**Authors:** Valerie Le Sage, Alessandro Cinti, Raquel Amorim, Andrew J. Mouland

**Affiliations:** 1 HIV-1 RNA Trafficking Laboratory, Lady Davis Institute at the Jewish General Hospital, Montréal, QC H3T 1E2, Canada; vle_sage@hotmail.com (V.L.S.); alessandro.cinti@mail.mcgill.ca (A.C.); raquel.amorim@mail.mcgill.ca (R.A.); 2 Department of Medicine, McGill University, Montréal, QC H3A 0G4, Canada

**Keywords:** PI3K, Akt, mTOR, virus, 4EBP1, autophagy

## Abstract

The mammalian target of rapamycin (mTOR) is a central regulator of gene expression, translation and various metabolic processes. Multiple extracellular (growth factors) and intracellular (energy status) molecular signals as well as a variety of stressors are integrated into the mTOR pathway. Viral infection is a significant stress that can activate, reduce or even suppress the mTOR signaling pathway. Consequently, viruses have evolved a plethora of different mechanisms to attack and co-opt the mTOR pathway in order to make the host cell a hospitable environment for replication. A more comprehensive knowledge of different viral interactions may provide fruitful targets for new antiviral drugs.

## 1. Introduction

Virus replication requires successful adaption to the host environment, which is achieved through co-opting cellular pathways, including nutrient, energy and macromolecular synthesis to drive the production of infectious particles. In the process of co-opting multiple cellular pathways to suit its needs, the virus triggers a host stress response that can lead to a global inhibition of protein synthesis to restrict consumption of nutrients and energy to promote cell survival.

The mammalian target of rapamycin (mTOR) is an evolutionarily conserved serine/threonine kinase and is a component of two functionally distinct protein complexes, mTORC1 and mTORC2. Each complex responds to diverse environmental cues and has different regulatory properties, as well as cellular activities. The adaptor proteins Raptor and Rictor are distinguishing components of mTORC1 and mTORC2, respectively [1]. Cap-dependent mRNA translation is regulated by mTORC1 via the phosphorylation of downstream effectors, being the eukaryotic initiation factor 4E (eIF4E)-binding protein 1 (4EBP1) and the p70 ribosomal S6 kinase 1 (S6K1). In nutrient-replete conditions, mTORC1 acts to trigger ribosome assembly and RNA translation so as to promote cell growth and proliferation, while suppressing autophagy. The upstream regulation of mTORC2 has not been fully defined, yet early evidence suggests that mTORC2 associates with the ribosome and that insulin-stimulated phosphatidylinositol 3-kinase (PI3K) signaling primarily increases mTORC2-ribosome binding [2]. Importantly, mTORC2 plays roles in cell survival and actin reorganization [3,4]. Rapamycin is a lipophilic macrolide isolated from bacteria, which specifically inhibits mTORC1 but not mTORC2 [5]. Rapamycin is current approved for clinical use and is a promising anti-cancer therapeutic agent.

Diverse stresses filter into the mTOR signaling network at various points in the pathway to influence and orchestrate a stress response. The tightly controlled, multistep process of activation begins with a cell surface receptor binding to its cognate ligand and transducing the signal to PI3K. For example, insulin activates PI3K to convert phosphoinositide (PI) 4,5-bisphosphate (PIP_2_) to PI 3,4,5-triphosphate (PIP_3_) [6]. Subsequently, the growth factor-activated kinase Akt is stimulated by phosphorylation at a site in the activation loop (Thr308) upon recruitment to the plasma membrane by the PI products of PI3K. Akt can also be phosphorylated on a second site in the hydrophobic motif (Ser473) by mTORC2 [7]. In turn, activated Akt phosphorylates the negative regulator TSC2 (tuberous sclerosis protein 2) and results in the dissociation of the TSC complex (TSC1 and TSC2) from the lysosome [8]. Rheb (Ras homolog enriched in brain) is a small guanosine triphosphatase (GTPase) that in its GTP-loaded state activates mTORC1, while the GAP (GTPase-activating protein) activity of TSC2 acts to inactivate mTORC1 by hydrolyzing Rheb-GTP to Rheb-GDP [9]. Upon dissolution of the TSC complex, Rheb-GTP is regenerated to activate mTORC1 in response to growth factors [8,10]. Amino acid availability is transduced to mTORC1 directly by the small GTPase heterodimers RagA/RagC and RagB/RagD (Ras-related GTPase), a process that, together with the Ragulator complex, occurs at the lysosome surface [8,11,12,13]. Growth factor deficiency, energy deficit, hypoxia, reactive oxygen species and DNA damage are all examples of inhibitory stresses that signal through the TSC complex [14,15].

During stress, autophagy is a major survival response that sequesters and degrades intracellular material, including damaged organelles and proteins or pathogens [16]. It is established that autophagy is directly suppressed through an mTORC1-dependent mechanism in response to nutrient starvation, while mTORC2 can indirectly upregulate autophagy gene expression through suppression of the transcription factor forkhead box O3 (FOXO3) via Akt [17,18]. Autophagy is a threat to many viruses because it can result in the degradation of viral proteins, while for other viruses the process of autophagy is beneficial and necessary for replication.

Virus-infected cells initiate the stress response by activating autophagy to eliminate the invading organism or by initiating apoptosis to limit virus spread. As activation of mTORC1 not only inhibits apoptosis, but also counteracts stress-induced autophagy, viruses have evolved to maintain a basal level of activity along the PI3K/Akt/mTOR pathway. This review will focus on the different strategies and points at which viruses subvert the mTORC1 signaling network (Table 1). Special emphasis is placed not only on the importance of controlling and promoting viral mRNA translation but also on modulating apoptosis and autophagy through the mTORC1 signaling pathway.

## 2. Stimulation of PI3K

PI3Ks are a family of lipid kinases that are divided into three classes based on their structure and substrate specificity. Class I PI3Ks phosphorylate PIP_2_ to produce PIP_3_ and act as major downstream effectors of receptor tyrosine kinases (RTKs) and G protein coupled receptors (GPCRs), which respond to growth factors and cytokines. The tumor suppressor PTEN (phosphatase and tensin homolog deleted from chromosome 10) and protein phosphatase 2 (PP2A) functionally antagonize the PI3K signaling pathway [73,74,75]. Several PI3K-specific inhibitors such as LY294002 and Wortmannin are available [76].

**Figure 1:**
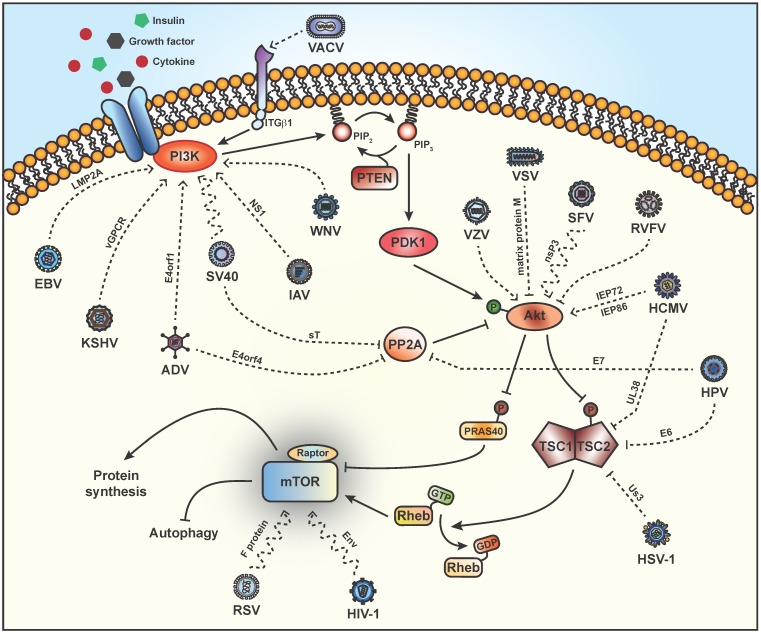
Schematic overview of viruses that subvert the PI3K/Akt/mTOR signaling pathway. External signals, such as growth factors, insulin and cytokines, activate phosphatidylinositol 3-kinase (PI3K) through receptor-mediated binding, which leads to phosphorylation of PIP_2_ into PIP_3_. mTORC1 is activated via a PI3K-dependent mechanism by Vaccinia virus (VACV), Epstein Barr virus (EBV), Kaposi’s sarcoma herpes virus (KSHV), Adenovirus (ADV), influenza A virus (IAV) and West Nile virus (WNV). Evidence suggests that simian virus 40 (SV40) phosphorylates Akt/mTOR potentially through PI3K. PIP_3_ recruits Akt to the plasma membrane whereby it is phosphorylated and activated by PDK1. Varicella zoster virus (VZV), Semliki Forest virus (SFV) and human cytomegalovirus (HCMV) are known to activate Akt by increasing phosphorylation, while vesicular stomatitis virus (VSV) and rift valley fever virus (RVFV) attenuate Akt signaling. Subsequently, activated Akt phosphorylates the negative regulator TSC2 (tuberous sclerosis protein 2), which results in the dissociation of the TSC complex (TSC1 and TSC2). Human papillomavirus (HPV) and HCMV activate mTORC1 by inhibiting or causing the degradation of TSC2, respectively. The activity of Akt is mimicked by herpes simplex virus type 1 (HSV-1), which causes the phosphorylation of TSC2. ADV, SV40 and HPV have dual activities and stimulate mTORC1 by blocking PP2A. Rheb (Ras homolog enriched in brain), in its GTP-loaded state, activates mTORC1, while TSC2 acts to inactivate mTORC1 by hydrolyzing Rheb-GTP to Rheb-GDP. Activation of mTORC1 enables continued protein synthesis and suppresses autophagy. Respiratory syncytial virus (RSV) and human immunodeficiency virus type 1 (HIV-1) have been shown to activate mTOR although the point at which these viruses attack the signaling pathway is unknown. Solid lines indicate the PI3K/Akt/mTOR signaling pathway. Dashed lines indicate clearly identified and wavy dashed lines represent ill-defined points at which viruses subvert the pathway.

Adenoviruses (ADV) cause a number of acute diseases and have an ability to transform cells, which makes them attractive vehicles for gene therapy. Early work indicates that activation of PI3K during ADV infection increases viral protein synthesis and virus production [22,77]. The major viral oncogenic determinant encoded by open reading frame 1 of early region 4 (*E4orf1*) is a small adaptor protein that associates with PDZ domain-containing proteins to act as a scaffold for the assembly of signaling complexes at the plasma membrane [78]. ADV E4orf1 mediates oncogenic cellular transformation, which is dependent on PI3K activation [79]. Mechanistically, E4orf1 forms a homotrimer with the cellular PDZ protein discs large 1 (Dlg1) and PI3K, which then translocate to the plasma membrane to induce activation of PI3K and increase translation [19,20,21] (Figure 1). In addition, E4orf4 stimulates mTORC1 via PP2A by inhibiting dephosphorylation of mTORC1, independently of TSC [22] (Figure 1).

The γ-herpesviruses, Epstein-Barr virus (EBV) and Kaposi’s sarcoma herpes virus (KSHV) respectively encode the latency protein LMP2A and the G protein-coupled receptor vGPCR, which activates the PI3K/Akt/mTOR pathway upstream of mTORC1 [23,35]. EBV LMP2A has been associated with nasopharyngeal carcinoma (NPC) [80] and constitutively activates mTOR growth regulatory pathways to mediate cell transformation via PI3K activation (Figure 1), as the PI3K inhibitor Wortmannin specifically blocked Akt phosphorylation in LMP2A-expressing cells [23]. Cancer spread has been linked to expression of EBV LMP2A on the cell surface as a result of increased expression of the metastatic tumor antigen 1 (MTA1) [81], which plays an important role in tumor recurrence and metastasis [82]. Ultimately, EBV appears to activate mTORC1 to increase MTA1 mRNA translation, as demonstrated by inhibition of 4EBP1 phosphorylation using the mTOR inhibitor, INK128 or knockdown of 4EBP1 that, respectively, decreased or increased the expression of MTA1 [81]. The authors propose that mTOR is a molecular hub linking LMP2A and MTA1-associated tumor malignancy and might be an interesting target in NPC treatment. Similarly, expression of KSHV vGPCR drives Kaposi’s sarcoma (KS) with activation of Akt occurring through PI3K-dependent as well as paracrine mechanisms [34,35] (Figure 1). Most recently, using a paracrine transformation model, Martin and colleagues show that the rapamycin-induced dephosphorylation of 4EBP1 results in decreased eIF4E-dependent mRNA translation and termination of KS development [83]. In agreement, studies have shown that treatment of renal-transplant patients with rapamycin causes tumor regression [84,85].

West Nile virus (WNV) is a member of the *Flaviviridae* family, which comprises other important human pathogens such as dengue virus (DENV), yellow fever virus and Japanese encephalitis virus (JEV). WNV infection increases mTOR activity through a PI3K-dependent mechanism (Figure 1), which is necessary for viral replication as evidenced by a detrimental effect on WNV growth after pharmacological inhibition of PI3K or mTOR [62,86]. mTORC1 activation is likely targeted by WNV to maintain translation of its positive-sense RNA genome [62] and delays WNV-induced apoptosis [63]. PI3K-dependent blocking of apoptosis has also been observed upon entry of DENV and JEV, although unlike WNV, PI3K pharmacological inhibition does not affect virus replication [64].

Murine polyomavirus (Py) and simian virus 40 (SV40) are small viruses of the *Polyomaviridae* family. The Py middle tumor antigen (MT) is bound to the plasma membrane, where it alters the activity of PI3K leading to phosphorylation of Akt and cell transformation [37,38,87]. In SV40 infection, Akt and mTOR are activated early, apparently through PI3K but as SV40 lacks a viral protein to inserts itself into the plasma membrane, the mechanism remains undefined and has yet to be followed up on [24,88] (Figure 1). Alternatively, the SV40 sT antigen has a PP2A interaction domain [41] that has been shown to activate Akt in a PP2A-dependent manner [42] (Figure 1).

**Figure 2:**
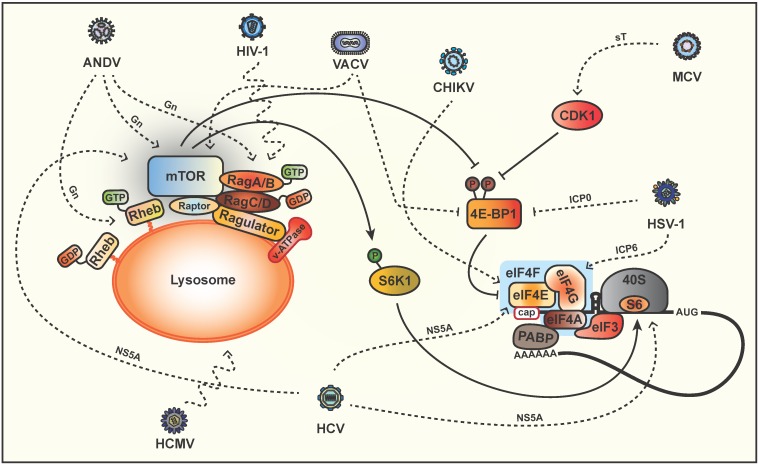
Downstream targets of mTORC1. Amino acid availability is transduced to mTORC1 directly by the small GTPase heterodimers RagA/RagC and RagB/RagD, a process that, together with the Ragulator complex, occurs at the lysosome surface. Human cytomegalovirus (HCMV) redistributes mTORC1 to a perinuclear localization in a dynein-dependent but Rag GTPase-independent mechanism. Andes virus (ANDV) modulates mTOR signaling at lysosomes and necessitates Rheb, RagA/B and LAMTOR1. The mTORC1 substrate, 4EBP1, is a negative regulator of mRNA translation initiation that binds to eIF4E to inhibit the formation of the eIF4F complex, which is made up of eIF4A, eIF4E and eIF4G. Phosphorylated 4EBP1 disassociates from eIF4E and frees it up to bind eIF4G, eIF3 and eIF4A to initiate cap-dependent translation. Chikungunya virus (CHIKV) increases the binding affinity of eIF4E to capped mRNA by increasing its phosphorylation. Merkel cell polyomavirus (MCV) causes CDK1-induced hyperphosphorylation of 4EBP1 to promote cap-dependent protein synthesis. Hepatitis C virus (HCV) interferes at multiple points in the pathway including activation of mTOR, enhanced eIF4F complex loading on mRNA and upregulation of internal ribosome entry site translation by associating with eIF4E and the 40S ribosome. Vaccinia virus (VACV) and Herpes simplex virus type 1 (HSV-1) are known to induce the degradation of 4EBP1 and affect the eIF4F complex. Solid lines indicate the PI3K/Akt/mTOR signaling pathway. Dashed lines indicate clearly identified and wavy dashed lines represent ill-defined points at which viruses subvert the pathway.

Old World alphavirus replication is not greatly affected by pharmacological inhibition of the mTOR pathway [60,89]. Semliki Forest virus (SFV) and Chikungunya virus (CHIKV) cause different diseases and pathology but both encode non-structural proteins (nsP) of which nsP3 is particularly interesting in terms of host-virus interactions. In SFV infected cells, Akt phosphorylation is observed to gradually increase over time at a point in the pathway upstream and/or at the level of Akt and is partially Wortmannin-insensitive [52] (Figure 1). Activation of Akt requires the hyperphosphorylated/acidic region of nsP3, which is attached to the plasma membrane as part of the viral replication complex upon internalization [52]. Evidence for CHIKV affecting the mTOR pathway is somewhat controversial with reports indicating a low level of activity [52], while other groups demonstrate activation of the PI3K/Akt/mTOR pathway [49]. A CHIKV-induced decrease in mTORC1 activity (at a point upstream of mTORC1) has also been reported, which correlates with an induction of autophagy, delayed apoptosis and enhanced CHIKV replication [50,51]. This information seems contradictory as CHIKV mRNA employs cap-dependent translation [90], yet upon rapamycin treatment CHIKV mRNA translation was enhanced with a tandem global reduction in cellular mRNA translation [50]. To bypass the inhibition of mTORC1 brought about by CHIKV infection, the virus appears to commandeer phosphorylated eIF4E (Figure 2) [91]. Sindbis virus (SINV) is another alphavirus that was found to suppress phosphorylation of Akt, mTOR, 4EBP1 and S6K1 in HEK cells at late times post-infection, suggesting that SINV replication blocks the mTOR pathway to modulate cell survival and protein synthesis [60]. However, in arthropod cells SINV was found to activate the mTOR pathway, which highlights the diverse replication strategies between vertebrates and arthropods [61].

The orthomyxovirus, influenza A virus (IAV) infects epithelial, lung and immune cells to finally result in cell death due to apoptosis [92,93]. IAV non-structural protein 1 (NS1) binds and activates PI3K [67,68] (Figure 1). Interestingly, inhibition of PI3K/Akt signaling was found to negatively impact viral RNA (vRNA) synthesis potentially as a result of a missing phosphorylation, either on a cellular factor involved in viral replication or of a viral protein [68]. IAV may have evolved to manipulate PI3K/Akt signaling at different phases of infection, but ultimately blockade of the PI3K/Akt pathway has detrimental effects on virus propagation [67,68,94]. Autophagy is triggered upon IAV infection [95], as a result of IAV control of mTOR, as demonstrated by the addition of Wortmannin [96]. IAV is a noteworthy case because blocking apoptosis still results in IAV-infected cell death due to massive autophagy activation driven by increased activity of mTORC1 and mTORC2/S6K1, whereas canonical autophagy is induced by transient PI3K activity and mTORC1 activation, even in IAV-infected cells undergoing apoptosis [96]. Canonical autophagy during acute infection may protect against immediate induction of cell death before the cells initiate apoptosis, which is necessary to complete the maturation of virus proteins [97]. Autophagy also appears to be important for earlier steps of IAV replication as autophagy deficiencies inhibit IAV vRNA and protein synthesis without affecting progeny virus production [98,99]. IAV highlights the viral need to strike a fine balance between the processes of apoptosis and autophagy.

Vaccinia virus (VACV) is a prototypical poxvirus that halts host protein synthesis to favor production of virus proteins. Prior to VACV entry, the virus clusters at plasma membrane lipid rafts and interacts with the raft-associated protein integrin β1 (ITGβ1), which mediates activation of PI3K/Akt and is necessary for subsequent virus endocytosis [43] (Figure 1). Pharmacological inhibition of the PI3K/Akt pathway during VACV infection significantly increases the induction of apoptosis and strongly suggests that subversion plays an important anti-apoptotic role to maintain high levels of virus replication [100]. Additionally, VACV also appears to cause the degradation of 4EBP1, as a consequence VACV alters the architecture of the eIF4F complex and causes a redistribution of eIF4E and eIF4G within viral factories, to facilitate viral replication [44].

PCV2 (porcine circovirus type 2) belongs to the *Circoviridae* family and is virulent in pigs [101]. Early and in the absence of active virus replication, PCV2 enhances phosphorylation of Akt in a PI3K-dependent manner, which is necessary to suppress premature apoptosis, promote viral DNA accumulation and protein synthesis [46]. Additionally, PCV2 induces autophagy by inhibiting mTOR signaling through a mechanism involving TSC2, ERK1/2 and AMPK [102,103].

## 3. Activation of Akt

PI3K activates its downstream effector, Akt by phosphorylation of T308 [104]. Akt, also known as protein kinase B, is a serine/threonine kinase that phosphorylates TSC2 to inhibit its GAP activity and activate mTORC1 [10]. mTORC2 can also regulate Akt by phosphorylation on a second site at residue S473 [7].

Human cytomegalovirus (HCMV), a β-herpesvirus, maintains mTORC1 activation regardless of cellular stress, which is important for the virus life cycle [105,106,107]. Akt phosphorylation is detected 96 hours after infection and requires expression of two HCMV immediate early proteins, IEP72 and IEP86 (Figure 1), which leads to an inhibition of apoptosis [24]. Varicella zoster virus (VZV) causes chickenpox and is example of a α-herpesvirus that causes a strong and rapid increase in phospho-Akt upon infection independent of PI3K phosphorylation [45] (Figure 1). Although phosphorylation of mTOR was detected in this study and likely has an effect on translation, it appears that subversion of PI3K/Akt signaling is important to antagonize virus-induced cell death [45].

The rabbit-specific poxvirus, Myxoma virus (MYXV) causes a lethal infection in rabbits. MYXV also appears to be able to infect human tumors *in vitro* but is only permissive in those cells expressing high levels of phosphorylated Akt [40]. In tumor cell lines with low levels of Akt activation, the host range factor M-T5 is necessary for MYXV replication and interacts with Akt to enhance its kinase activity [39,40]. Replication of a M-T5 deficient MYXV strain can be rescued by addition a PP2A-specific inhibitor to maintain phosphorylated Akt [108] and in non-permissive human tumor cells rapamycin appears to, in the context of virus infection, enhance Akt activity and increase virus spread [109].

By contrast, there are a number of viruses that suppress Akt activation. Vesicular stomatitis virus (VSV) causes dephosphorylation of Akt through the viral matrix protein M (Figure 1), which cannot be overcome by constitutive targeting of Akt to the plasma membrane or accumulation of PIP_3_ [72]. Infection with VSV halts host mRNA translation without impairing its own viral protein synthesis. The block in host translation temporally coincides with the VSV-induced dephosphorylation of eIF4E and 4EBP1 via a mechanism that has yet to be elucidated [110]. Inhibition of Akt phosphorylation by Coxsackievirus A16 (CA16) contributes to the upregulation of autophagy, which enhances viral replication [53]. Avian reovirus (ARV) protein p17 activates PTEN, which in turn prevents Akt activation and host cellular translational shutoff [47]. Early upon infection by the avibirnavirus infectious bursal disease virus (IBDV), the VP2 capsid protein inactivates Akt to stimulate autophagy [48].

Rift Valley virus (RVFV) enforces a host translational arrest through interplay with mTOR signaling [111]. This member of the *Bunyaviridae* family is an interesting case as infection attenuates Akt signaling (Figure 1) resulting in dephosphorylation of 4EBP1 [71], the timing of which coincides with a significant degradation of 5′-TOP (terminal oligopyrimidine) mRNA and specifically requires 4EBP1 [111]. By shunting 5′-TOP mRNA to P bodies for decay RVFV is able to utilize cap-snatching machinery for its own viral translation [111].

The *Paramyxoviridae* family includes a number of important human pathogens including mumps, measles virus (MV) and respiratory syncytial virus (RSV). MV acts to downregulate Akt activity, therefore limiting the cell’s immune response to infection [69,112]. RSV causes severe infections in infants, immunocompromised individuals and the elderly, in part because RSV infection does not result in the development of protective immunity [113]. mTOR is a major regulator of memory CD8+ T cell differentiation [114] and acute RSV infection appears to suppress memory CD8+ T cell activity through the phosphorylation of mTOR *in vitro* and *in vivo* (in infants infected with RSV) [70]. RSV F protein is capable of inducing mTOR phosphorylation in the absence of virus replication (Figure 1), which is inhibited by rapamycin but not LY294002, indicating a PI3K-independent mechanism [70,115]. Activation of mTOR could potentially occur via a cell surface receptor as some are known to interact with RSV F protein [70].

## 4. TSC2

Downstream of Akt, the TSC complex is formed by an obligate heterodimer and regulates mTOR activation through the GAP activity of TSC2, which changes active, GTP-bound Rheb into an inactive GDP-bound state [116].

Human papillomavirus (HPV) is a member of the *Polyomaviridae* family that causes squamous cell carcinoma in a range of different tissue types, including cervical and head and neck cancers. The HPV-16 E6 and E7 oncoproteins activate the PI3K/Akt/mTOR signaling pathway to affect tumor initiation and progression (reviewed in [117]). Several studies indicate that HPV protein E6 mediates the proteasome-dependent degradation of TSC2 to activate mTORC1 [28,29] (Figure 1), while another group determined that cap-dependent translation was enhanced by HPV protein E6 through the activation of Akt via PDK1 and mTORC2 [118]. Similar to other members in the *Polyomaviridae* family, HPV protein E7 can interact with PP2A and interfere with the dephosphorylation and inhibition of Akt [30] (Figure 1).

Specific to β-herpesviruses, HCMV UL38 is a multifunctional protein that blocks apoptosis [119,120] and inhibits TSC2 activation of mTORC1 [26] (Figure 1). Interestingly, Bai and colleagues have defined the interacting domain of UL38 to TSC2 and found that a TSC2-binding deficient UL38 mutant was still able to maintain mTORC1 activation [121]. This result was confirmed by TSC2 knockdown whereby UL38 increased mTORC1 activity and point toward redundant mechanisms of mTORC1 upregulation by HCMV to maintain viral protein synthesis and replication [121]. Recently, mTOR has been shown to play a role in the switch from HCMV latency. In latently infected hematopoietic cells, the HCMV viral genome is suppressed as a consequence of the binding of KAP1 and two other host proteins, which is abrogated upon phosphorylation by mTOR to reactivate the virus [122].

Herpes simplex virus type 1 (HSV-1) is an α-herpesvirus that undergoes productive replication at the site of infection and persist in neurons in a latent state. At early times post-infection, HSV-1 appears to transiently activate Akt [123], however, upon addition of Akt inhibitors mTORC1 signaling was sustained [31]. Us3 is a serine/threonine kinase that was later shown to be responsible for this activity and acts as an Akt mimic to directly phosphorylate TSC2 (Figure 1), thus inactivating 4EBP1 in infected cells [31,124]. Interestingly, Us3 shares no sequence homology with Akt and therefore is not affected by pharmacological drugs that limit Akt activity [31]. Regardless of the mechanism, encoding Us3 (unique to α-herpesviruses) or a TSC2-binding protein (β-herpesviruses) signifies an important mTORC1 regulatory intersection that is critical and must be antagonized by different herpesviruses.

## 5. Phosphorylation of Downstream Targets

The cell can instantly adjust to changing environmental conditions by regulation mRNA translation. Modulation of mTOR is important for viruses with an RNA genome as they must compete directly with host mRNAs for access to translational components and to oppose the stress response, thus maintaining cap-dependent translation. The mTORC1 substrate, 4EBP1, is a negative regulator of mRNA translation initiation that binds to eIF4E to inhibit the formation of the eIF4F complex, which is made up of eIF4A, eIF4E and eIF4G. Phosphorylated 4EBP1 disassociates from eIF4E and frees it up to bind eIF4G, eIF3 and eIF4A to initiate cap-dependent translation [125]. A comprehensive review on mechanism that viruses use to co-opt eIF4E was recently published [126] so in the following section, we will focus on manipulation of cap-dependent translation by 4EBP1.

In addition to modulating mTOR activity by targeting PI3K, Akt or TSC2, many herpesviruses also act downstream of the mTOR node. Different families of herpesviruses enhance eIF4F assembly, by stimulating phosphorylation of eIF4E and 4EBP1 [25,32,127]. During HCMV infection host protein synthesis is maintained whereas it is strongly inhibited by HSV-1 [128]. This difference may stem, at least in part, from the HSV-1 ICP0-dependent proteosomal degradation of 4EBP1 (Figure 2), as opposed to HCMV, which induces the accumulation of eIF4E, eIF4G and PABP [25,32]. Interestingly, HSV-1 ICP6 associates with eIF4G and plays a necessary role as a chaperone to promote active eIF4F complex assembly [33] (Figure 2).

The recently discovered Merkel cell polyomavirus (MCV) causes human skin cancer Merkel cell carcinoma [129] and encodes a small T (sT) antigen that is important for oncogenesis [130]. In an mTOR-independent mechanism, MCV sT causes cyclin-dependent kinase 1 (CDK1)-induced hyperphosphorylation of 4EBP1 by acting as a promiscuous E3 ligase inhibitor to promote cap-dependent protein synthesis [36,131] (Figure 2). This is in sharp contrast to the hypophosphorylation of 4EBP1 that is observed late in infection by SV40 and requires the PP2A binding region of sT [132] (Figure 1). SV40 late structural protein expression is driven by an internal ribosome entry site (IRES) and is therefore unaffected by virus-induced inhibition of cap-dependent translation [133].

Hepatitis C virus (HCV) is another member of the *Flaviviridae* family, which causes chronic infection and hepatocellular carcinoma. HCV non-structural protein 5A (NS5A) is a multifunctional protein that enhances phosphorylation of mTOR but also appears to enhance transcription of 4EBP and S6K mRNAs, which are both phosphorylated to allow cap-dependent translation initiation of a specific set of transcripts [55]. In addition to affecting the downstream effectors of mTOR, HCV also interferes at multiple points in the pathway with contradictory studies arguing the mechanism of mTOR activation. It has been proposed that NS5A can directly activate PI3K/Akt signaling by directly binding PI3K [56,58] while other studies indicate that NS5A activates mTOR independent of PI3K and Akt activation. The first suggests direct binding of NS5A to the mTORC1 cofactor FKBP38 (FK506-binding protein 38) to block apoptosis [57] (Figure 2), whereas the second proposes differential regulation of Akt through upregulation of mTORC2 [55]. Activation of the mTORC1 pathway by HCV has been linked to anti-apoptotic signals that ensure cell survival and maintain persistence by promoting steady state levels of virus replication [134]. In another study, Bose and colleagues describe a mechanism whereby HCV downregulates TSC1/2 expression and subsequently activation of mTOR and S6K1 [54]. It was hypothesized that NS5A activates cap-dependent translation by enhanced eIF4F complex loading to augment cell proliferation and tumorigenesis [55] (Figure 2). Most recently, HCV NS5A has been shown to associate with eIF4E and 40S ribosomes to form a complex that upregulates HCV IRES translation [135] (Figure 2). HCV establishes a persistent infection in hepatocytes through the induction of endoplasmic reticulum (ER) stress, which goes on to inhibit Akt and downregulate mTORC1 in order to activate autophagy [136]. Evidence indicates that autophagy is induced by HCV through the transcriptional upregulation of Beclin-1, in a context in which also mTOR is activated by the virus [137]. As suggested by the authors, a potential explanation of this paradox is likely that HCV infection is inducing autophagy for establishment of infection, while activating mTOR signaling for hepatocyte growth. Indeed, HCV-induced autophagy is required for virus replication [138,139]. As for other *Flaviviridae* members, Zika virus enhances autophagy to increase replication [140], whereas WNV replication is independent of autophagy [86,141].

## 6. Modulation of mTOR via Lysosomal Signaling

Activation of mTORC1 by amino acid starvation is independent from TSC2/Rheb axis and regulated by Rag GTPases. RagA/RagC and RagB/RagD form constitutive heterodimers, where in the presence of amino acids RagA and RagB are GTP loaded and therefore augment their affinity to Raptor [142,143]. Rag-bound mTORC1 is relocalized to late endosomal/lysosomal surfaces, thus bringing the complex into close proximity to Rheb [13,143].

HCMV infection is able to maintain mTORC1 activation in the presence of various types of stress that signal through the TSC complex, as described in the previous sections. However, HCMV is also able to block stress, such as oxidative stress and amino acid starvation that operate via a mechanism that does not depend on the binding of UL38 to TSC [105,107]. Very early upon infection, before synthesis of UL38, HCMV redistributes mTORC1 to a perinuclear localization in a dynein-dependent but Rag GTPase-independent mechanism [27] (Figure 2). This distribution is maintained during amino acid depletion and corresponds to constitutive mTORC1 activity, as mTOR colocalizes with its activator Rheb [27,105].

Andes virus (ANDV) is a highly lethal hantavirus that causes hypoxia and acute pulmonary edema [144,145]. ANDV infection activates mTOR and increased phosphorylation of S6K1 [65]. Rapamycin inhibited mTOR signaling with no apparent effect on ANDV titers, instead appeared to enhance monolayer permeability and hinder giant endothelial cell formation [65]. In contrast, McNulty and colleagues do not observe S6K1 phosphorylation, rather they show that a rapamycin analog (temsirolimus) reduces ANDV protein expression and replication without affecting host protein synthesis [66]. In infected cells, the ANDV glycoprotein Gn colocalizes with mTOR and lysosomes and necessitates the mTORC1 lysosomal activators (Rheb, RagA/B and LAMTOR1) [66] (Figure 2), which suggests that ANDV modulates mTOR signaling at lysosomes.

Human immunodeficiency virus type 1 (HIV-1) is a characterized by persistent virus replication, widespread inflammation and massive CD4+ T cell depletion. Mounting evidence indicates that autophagy is a critical target for HIV-1 during virus replication [146,147] but little is known about regulation of autophagy through mTORC1 by HIV-1. In dendritic cells, HIV-1 envelope (Env) glycoprotein induces activation of mTOR and S6K1, leading to inhibition of autophagy and increased virus infection (Figure 1). Accordingly, treatment of HIV-1-infected cells with rapamycin decreased the spread of virus infection [59]. In macrophages, Campbell and colleagues demonstrated that during permissive infection, Nef binds to Beclin-1 and retains TFEB in the cytoplasm to inhibit autophagy [148]. Upon mTOR inhibition, TFEB is dephosphorylated and translocated to the nucleus where it increases autophagy and lysosomal gene expression. This lead the authors to speculate that Nef-Beclin-1 binding increases mTOR activation via TLR signaling complex disruption with subsequent TFEB phosphorylation, cytoplasmic sequestration and inhibition of autophagy [148]. Our group has recently shown that HIV-1 is also able to activate mTORC1 at a late stage of virus replication although through a yet unknown point in the pathway likely at or upstream of PI3K, as mTOR or PI3K inhibitors specifically blocked HIV-1-induced activation of mTORC1 [149]. Interestingly, HIV-1 is unable to maintain mTORC1 activation in the absence of nutrients (amino acids and glucose), but is capable of redistributing mTOR-associated late endosomes/lysosomes to the cell periphery through a mechanism reliant on the small Rag GTPases A and B [149] (Figure 2). Finally, drugs that modify the mTORC1 signaling pathway also alter HIV-1 replication [150,151,152].

## 7. Conclusions

As a master regulator of diverse cell functions, viruses target mTOR as a strategy to ensure their replication. Establishment of latent or chronic infection induces cellular transformation and oncogenesis occurs as a consequence of subverting the mTORC1 pathway to promote cell survival and a resistance to apoptosis. PI3K and Akt activation or TSC complex inactivation are typical upstream points of attack for numerous viruses including EBV [23,81], HPV [117] and ADV [77,79] whereas other viruses favor downstream targets, such HCV [55]. Illustrating the importance of this pathway in virus replication, several viruses employ multi-pronged strategies to subvert mTOR signaling. For example, HCMV activates Akt [24] and inhibits TSC2 [26] to strongly maintain 4EBP1 phosphorylation regardless of imposed stress conditions to allow continued viral protein synthesis [127]. This raises the question as to whether other viruses have control over mTOR through multiple interactions that have yet to be described.

The cellular stress imposed by an acute infection requires that the virus commandeer mTOR signaling to sustain protein synthesis, which is the case for WNV [62], SINV [60] and VSV [110]. Overcoming the host cellular stress response by blocking apoptosis and modulating autophagy is important for IAV [96], CHIKV [51] and CA16 [53]. As the knowledge in this field is relatively recent, new research is expected to elaborate on the crosstalk and clarify the controversies of virus modulation of apoptosis and autophagy through the mTOR signaling pathway.

In this review, we highlight different mechanisms that viruses employ to subvert the mTORC1 pathway to favor replication. Understanding the relationship between viral replication and mTOR signaling may provide a basis for developing new antiviral drugs. Numerous cancer cells depend on the mTOR pathway for efficient cellular proliferation. Preclinical studies and clinical trials for the use of mTORC1 inhibitors such as rapamycin are an important avenue of research in the fight against cancers, as mTOR signaling is highly active in a number of different malignancies. Moreover, ongoing research for new inhibitors of mTOR may increase the therapeutic arsenal of available drugs and strengthen the current knowledge of the regulation of mTOR over cellular metabolism. As many viruses hijack the mTOR pathway to favor replication, using mTOR inhibitors as monotherapy or together with targeted antiviral drugs as a new strategy to treat viral infections may provide benefits in the clinic. However, the diverse avenues of viral attack within the mTOR pathway, most of them not yet completely understood, as well as unforeseen side effects related to the immunosuppressant activity of mTOR inhibitors may be a challenge in this development. Unveiling key interactions and clearly defining viral dependencies are crucial to the rise of targeted mTOR antiviral therapy.

## Figures and Tables

**Table 1:** Viruses that target the mammalian target of rapamycin (mTOR) signaling pathway.

Group Classification	Virus	Viral Protein	Target	Reference
dsDNA	Adenovirus	E4orf1	PI3K activation	[19,20,21]
dsDNA	Adenovirus	E4orf4	Blocks dephosphorylation of mTORC1 via PP2A	[22]
dsDNA	Epstein-Barr virus	LMP2A	PI3K activation	[23]
dsDNA	Human cytomegalovirus	IEP72 and IEP86	Activates Akt	[24]
dsDNA	Human cytomegalovirus	N/D	Increase in abundance of eIF4F complex proteins	[25]
dsDNA	Human cytomegalovirus	UL38	Binds and antagonizes TSC2	[26]
dsDNA	Human cytomegalovirus	N/D	Redistribution of mTORC1 to a perinuclear localization	[27]
dsDNA	Human papillomavirus	protein E6	Causes degradation of TSC2	[28,29]
dsDNA	Human papillomavirus	protein E7	Inhibits dephosphorylation of Akt through an interaction with PP2A	[30]
dsDNA	Herpes simplex virus type 1	Us3	Akt mimic	[31]
dsDNA	Herpes simplex virus type 1	ICP0	Degradation of 4EBP1 by the proteasome	[32]
dsDNA	Herpes simplex virus type 1	ICP6	Associates with eIF4G	[33]
dsDNA	Kaposi’s Sarcoma Herpesvirus	vGPCR	PI3K activation	[34,35]
dsDNA	Merkel cell polyomavirus	sT	Hyperphosphorylation of 4EBP1	[36]
dsDNA	Murine polyomavirus	MT	PI3K activation	[37,38]
dsDNA	Myxoma virus	M-T5	Activates Akt	[39,40]
dsDNA	Simian virus 40	sT	Activates Akt through an interaction with PP2A	[41,42]
dsDNA	Vaccinia virus	N/D	Mediates activation of PI3K/Akt through protein integrin β1 (ITGβ1)	[43]
dsDNA	Vaccinia virus	N/D	Alters architecture of eIF4F complex	[44]
dsDNA	Varicella zoster virus	pORFs 47 and 66	Activates Akt	[45]
ssDNA	Porcine circovirus type 2	N/D	Activates PI3K	[46]
dsRNA	Avian reovirus	Protein p17	Inactivation of Akt through activation of PTEN	[47]
dsRNA	Infectious bursal disease virus	VP2 capsid	Inactivates Akt	[48]
+ssRNA	Chikungunya virus	N/D	Controversial activation or suppression of PI3K/Akt/mTOR pathway	[49,50,51,52]
+ssRNA	Coxsackievirus A16	N/D	Inhibits Akt phosphorylation	[53]
+ssRNA	Hepatitis C virus	NS5A	Activation of PI3K/Akt/mTOR pathway	[54,55,56,57,58]
+ssRNA	Human immunodeficiency virus type 1	Env	Activation of mTOR	[59]
+ssRNA	Semliki Forest virus	nsP3	Activation of Akt	[52]
+ssRNA	Sindbis virus	N/D	Suppression in HEK cells and activation in arthropod cells of PI3K/Akt/mTOR pathway	[60,61]
+ssRNA	West Nile virus, Dengue virus, Japanese encephalitis virus	N/D	PI3K activation	[62,63,64]
−ssRNA	Andes virus	Gn	Modulation of mTOR and lysosomal signaling	[65,66]
−ssRNA	Influenza A virus	NS1	Activates PI3K	[67,68]
−ssRNA	Measles virus	N/D	Inactivates Akt	[69]
−ssRNA	Respiratory syncytial virus	F protein	Induces phosphorylation of mTOR via a PI3K-independent mechanism	[70]
−ssRNA	Rift Valley virus	N/D	Inhibits Akt phosphorylation	[71]
−ssRNA	Vesicular stomatitis virus	Matrix protein M	Inactivates Akt	[72]

N/D: not determined; dsDNA: double stranded DNA; dsRNA: double stranded RNA; +ssRNA: positive single-stranded RNA; -ssRNA: negative single-stranded RNA; PI3K: phosphatidylinositol 3-kinase; mTOR; mammalian target of rapamycin; mTORC: mTOR complex; PP2A: protein phosphatase 2; PTEN: phosphatase and tensin homolog deleted from chromosome 10; 4EBP1: eukaryotic initiation factor 4E (eIF4E)-binding protein 1.

## References

[1] Foster K.G., Fingar D.C. (2010). Mammalian target of rapamycin (mTOR): Conducting the cellular signaling symphony. J. Biol. Chem..

[2] Zinzalla V., Stracka D., Oppliger W., Hall M.N. (2011). Activation of mTORC2 by association with the ribosome. Cell.

[3] Sarbassov D.D., Ali S.M., Kim D.H., Guertin D.A., Latek R.R., Erdjument-Bromage H., Tempst P., Sabatini D.M. (2004). Rictor, a novel binding partner of mTOR, defines a rapamycin-insensitive and raptor-independent pathway that regulates the cytoskeleton. Curr. Biol..

[4] Jacinto E., Loewith R., Schmidt A., Lin S., Ruegg M.A., Hall A., Hall M.N. (2004). Mammalian TOR complex 2 controls the actin cytoskeleton and is rapamycin insensitive. Nat. Cell Biol..

[5] Guertin D.A., Sabatini D.M. (2009). The pharmacology of mTOR inhibition. Sci. Signal..

[6] Fruman D.A., Meyers R.E., Cantley L.C. (1998). Phosphoinositide kinases. Annu. Rev. Biochem..

[7] Sarbassov D.D., Guertin D.A., Ali S.M., Sabatini D.M. (2005). Phosphorylation and regulation of Akt/PKB by the rictor-mTOR complex. Science.

[8] Menon S., Dibble C.C., Talbott G., Hoxhaj G., Valvezan A.J., Takahashi H., Cantley L.C., Manning B.D. (2014). Spatial control of the TSC complex integrates insulin and nutrient regulation of mTORC1 at the lysosome. Cell.

[9] Inoki K., Li Y., Xu T., Guan K.L. (2003). Rheb GTPase is a direct target of TSC2 GAP activity and regulates mTOR signaling. Genes Dev..

[10] Inoki K., Li Y., Zhu T., Wu J., Guan K.L. (2002). TSC2 is phosphorylated and inhibited by Akt and suppresses mTOR signalling. Nat. Cell Biol..

[11] Efeyan A., Sabatini D.M. (2013). Nutrients and growth factors in mTORC1 activation. Biochem. Soc. Trans..

[12] Bar-Peled L., Schweitzer L.D., Zoncu R., Sabatini D.M. (2012). Ragulator is a GEF for the rag GTPases that signal amino acid levels to mTORC1. Cell.

[13] Sancak Y., Bar-Peled L., Zoncu R., Markhard A.L., Nada S., Sabatini D.M. (2010). Ragulator-Rag complex targets mTORC1 to the lysosomal surface and is necessary for its activation by amino acids. Cell.

[14] Wang X., Proud C.G. (2011). mTORC1 signaling: What we still don’t know. J. Mol. Cell Biol..

[15] Huang J., Manning B.D. (2009). A complex interplay between Akt, TSC2 and the two mTOR complexes. Biochem. Soc. Trans..

[16] Levine B., Mizushima N., Virgin H.W. (2011). Autophagy in immunity and inflammation. Nature.

[17] Brunet A., Bonni A., Zigmond M.J., Lin M.Z., Juo P., Hu L.S., Anderson M.J., Arden K.C., Blenis J., Greenberg M.E. (1999). Akt promotes cell survival by phosphorylating and inhibiting a Forkhead transcription factor. Cell.

[18] Mammucari C., Milan G., Romanello V., Masiero E., Rudolf R., Del Piccolo P., Burden S.J., Di Lisi R., Sandri C., Zhao J. (2007). FoxO3 controls autophagy in skeletal muscle *in vivo*. Cell Metab..

[19] Frese K.K., Latorre I.J., Chung S.H., Caruana G., Bernstein A., Jones S.N., Donehower L.A., Justice M.J., Garner C.C., Javier R.T. (2006). Oncogenic function for the Dlg1 mammalian homolog of the Drosophila discs-large tumor suppressor. EMBO J..

[20] Kong K., Kumar M., Taruishi M., Javier R.T. (2014). The human adenovirus E4-ORF1 protein subverts discs large 1 to mediate membrane recruitment and dysregulation of phosphatidylinositol 3-kinase. PLoS Pathog..

[21] Kumar M., Kong K., Javier R.T. (2014). Hijacking Dlg1 for oncogenic phosphatidylinositol 3-kinase activation in human epithelial cells is a conserved mechanism of human adenovirus E4-ORF1 proteins. J. Virol..

[22] O’Shea C., Klupsch K., Choi S., Bagus B., Soria C., Shen J., McCormick F., Stokoe D. (2005). Adenoviral proteins mimic nutrient/growth signals to activate the mTOR pathway for viral replication. EMBO J..

[23] Moody C.A., Scott R.S., Amirghahari N., Nathan C.O., Young L.S., Dawson C.W., Sixbey J.W. (2005). Modulation of the cell growth regulator mTOR by Epstein-Barr virus-encoded LMP2A. J. Virol..

[24] Yu Y., Alwine J.C. (2002). Human cytomegalovirus major immediate-early proteins and simian virus 40 large T antigen can inhibit apoptosis through activation of the phosphatidylinositide 3'-OH kinase pathway and the cellular kinase Akt. J. Virol..

[25] Walsh D., Perez C., Notary J., Mohr I. (2005). Regulation of the translation initiation factor eIF4F by multiple mechanisms in human cytomegalovirus-infected cells. J. Virol..

[26] Moorman N.J., Cristea I.M., Terhune S.S., Rout M.P., Chait B.T., Shenk T. (2008). Human cytomegalovirus protein UL38 inhibits host cell stress responses by antagonizing the tuberous sclerosis protein complex. Cell Host Microbe.

[27] Clippinger A.J., Alwine J.C. (2012). Dynein mediates the localization and activation of mTOR in normal and human cytomegalovirus-infected cells. Genes Dev..

[28] Lu Z., Hu X., Li Y., Zheng L., Zhou Y., Jiang H., Ning T., Basang Z., Zhang C., Ke Y. (2004). Human papillomavirus 16 E6 oncoprotein interferences with insulin signaling pathway by binding to tuberin. J. Biol. Chem..

[29] Zheng L., Ding H., Lu Z., Li Y., Pan Y., Ning T., Ke Y. (2008). E3 ubiquitin ligase E6AP-mediated TSC2 turnover in the presence and absence of HPV16 E6. Genes Cells.

[30] Pim D., Massimi P., Dilworth S.M., Banks L. (2005). Activation of the protein kinase B pathway by the HPV-16 E7 oncoprotein occurs through a mechanism involving interaction with PP2A. Oncogene.

[31] Chuluunbaatar U., Roller R., Feldman M.E., Brown S., Shokat K.M., Mohr I. (2010). Constitutive mTORC1 activation by a herpesvirus Akt surrogate stimulates mRNA translation and viral replication. Genes Dev..

[32] Walsh D., Mohr I. (2004). Phosphorylation of eIF4E by Mnk-1 enhances HSV-1 translation and replication in quiescent cells. Genes Dev..

[33] Walsh D., Mohr I. (2006). Assembly of an active translation initiation factor complex by a viral protein. Genes Dev..

[34] Montaner S., Sodhi A., Pece S., Mesri E.A., Gutkind J.S. (2001). The Kaposi’s sarcoma-associated herpesvirus G protein-coupled receptor promotes endothelial cell survival through the activation of Akt/protein kinase B. Cancer Res..

[35] Sodhi A., Chaisuparat R., Hu J., Ramsdell A.K., Manning B.D., Sausville E.A., Sawai E.T., Molinolo A., Gutkind J.S., Montaner S. (2006). The TSC2/mTOR pathway drives endothelial cell transformation induced by the Kaposi’s sarcoma-associated herpesvirus G protein-coupled receptor. Cancer Cell.

[36] Shuda M., Kwun H.J., Feng H., Chang Y., Moore P.S. (2011). Human Merkel cell polyomavirus small T antigen is an oncoprotein targeting the 4E-BP1 translation regulator. J. Clin. Investig..

[37] Dahl J., Jurczak A., Cheng L.A., Baker D.C., Benjamin T.L. (1998). Evidence of a role for phosphatidylinositol 3-kinase activation in the blocking of apoptosis by polyomavirus middle T antigen. J. Virol..

[38] Utermark T., Schaffhausen B.S., Roberts T.M., Zhao J.J. (2007). The p110alpha isoform of phosphatidylinositol 3-kinase is essential for polyomavirus middle T antigen-mediated transformation. J. Virol..

[39] Werden S.J., Barrett J.W., Wang G., Stanford M.M., McFadden G. (2007). M-T5, the ankyrin repeat, host range protein of myxoma virus, activates Akt and can be functionally replaced by cellular PIKE-A. J. Virol..

[40] Wang G., Barrett J.W., Stanford M., Werden S.J., Johnston J.B., Gao X., Sun M., Cheng J.Q., McFadden G. (2006). Infection of human cancer cells with myxoma virus requires Akt activation via interaction with a viral ankyrin-repeat host range factor. Proc. Natl. Acad. Sci. USA.

[41] Pallas D.C., Shahrik L.K., Martin B.L., Jaspers S., Miller T.B., Brautigan D.L., Roberts T.M. (1990). Polyoma small and middle T antigens and SV40 small t antigen form stable complexes with protein phosphatase 2A. Cell.

[42] Rodriguez-Viciana P., Collins C., Fried M. (2006). Polyoma and SV40 proteins differentially regulate PP2A to activate distinct cellular signaling pathways involved in growth control. Proc. Natl. Acad. Sci. USA.

[43] Izmailyan R., Hsao J.C., Chung C.S., Chen C.H., Hsu P.W., Liao C.L., Chang W. (2012). Integrin beta1 mediates vaccinia virus entry through activation of PI3K/Akt signaling. J. Virol..

[44] Walsh D., Arias C., Perez C., Halladin D., Escandon M., Ueda T., Watanabe-Fukunaga R., Fukunaga R., Mohr I. (2008). Eukaryotic translation initiation factor 4F architectural alterations accompany translation initiation factor redistribution in poxvirus-infected cells. Mol. Cell. Biol..

[45] Rahaus M., Desloges N., Wolff M.H. (2007). Varicella-zoster virus requires a functional PI3K/Akt/GSK-3alpha/beta signaling cascade for efficient replication. Cell. Signal..

[46] Wei L., Zhu S., Wang J., Liu J. (2012). Activation of the phosphatidylinositol 3-kinase/Akt signaling pathway during porcine circovirus type 2 infection facilitates cell survival and viral replication. J. Virol..

[47] Huang W.R., Chiu H.C., Liao T.L., Chuang K.P., Shih W.L., Liu H.J. (2015). Avian Reovirus Protein p17 Functions as a Nucleoporin Tpr Suppressor Leading to Activation of p53, p21 and PTEN and Inactivation of PI3K/AKT/mTOR and ERK Signaling Pathways. PLoS ONE.

[48] Hu B., Zhang Y., Jia L., Wu H., Fan C., Sun Y., Ye C., Liao M., Zhou J. (2015). Binding of the pathogen receptor HSP90AA1 to avibirnavirus VP2 induces autophagy by inactivating the AKT-MTOR pathway. Autophagy.

[49] Das I., Basantray I., Mamidi P., Nayak T.K., Pratheek B.M., Chattopadhyay S., Chattopadhyay S. (2014). Heat shock protein 90 positively regulates Chikungunya virus replication by stabilizing viral non-structural protein nsP2 during infection. PLoS ONE.

[50] Joubert P.E., Stapleford K., Guivel-Benhassine F., Vignuzzi M., Schwartz O., Albert M.L. (2015). Inhibition of mTORC1 Enhances the Translation of Chikungunya Proteins via the Activation of the MnK/eIF4E Pathway. PLoS Pathog..

[51] Joubert P.E., Werneke S.W., de la Calle C., Guivel-Benhassine F., Giodini A., Peduto L., Levine B., Schwartz O., Lenschow D.J., Albert M.L. (2012). Chikungunya virus-induced autophagy delays caspase-dependent cell death. J. Exp. Med..

[52] Thaa B., Biasiotto R., Eng K., Neuvonen M., Gotte B., Rheinemann L., Mutso M., Utt A., Varghese F., Balistreri G. (2015). Differential Phosphatidylinositol-3-Kinase-Akt-mTOR Activation by Semliki Forest and Chikungunya Viruses Is Dependent on nsP3 and Connected to Replication Complex Internalization. J. Virol..

[53] Shi Y., He X., Zhu G., Tu H., Liu Z., Li W., Han S., Yin J., Peng B., Liu W. (2015). Coxsackievirus A16 elicits incomplete autophagy involving the mTOR and ERK pathways. PLoS ONE.

[54] Bose S.K., Shrivastava S., Meyer K., Ray R.B., Ray R. (2012). Hepatitis C virus activates the mTOR/S6K1 signaling pathway in inhibiting IRS-1 function for insulin resistance. J. Virol..

[55] George A., Panda S., Kudmulwar D., Chhatbar S.P., Nayak S.C., Krishnan H.H. (2012). Hepatitis C virus NS5A binds to the mRNA cap-binding eukaryotic translation initiation 4F (eIF4F) complex and up-regulates host translation initiation machinery through eIF4E-binding protein 1 inactivation. J. Biol. Chem..

[56] He Y., Nakao H., Tan S.L., Polyak S.J., Neddermann P., Vijaysri S., Jacobs B.L., Katze M.G. (2002). Subversion of cell signaling pathways by hepatitis C virus nonstructural 5A protein via interaction with Grb2 and P85 phosphatidylinositol 3-kinase. J. Virol..

[57] Peng L., Liang D., Tong W., Li J., Yuan Z. (2010). Hepatitis C virus NS5A activates the mammalian target of rapamycin (mTOR) pathway, contributing to cell survival by disrupting the interaction between FK506-binding protein 38 (FKBP38) and mTOR. J. Biol. Chem..

[58] Street A., Macdonald A., Crowder K., Harris M. (2004). The Hepatitis C virus NS5A protein activates a phosphoinositide 3-kinase-dependent survival signaling cascade. J. Biol. Chem..

[59] Blanchet F.P., Moris A., Nikolic D.S., Lehmann M., Cardinaud S., Stalder R., Garcia E., Dinkins C., Leuba F., Wu L. (2010). Human immunodeficiency virus-1 inhibition of immunoamphisomes in dendritic cells impairs early innate and adaptive immune responses. Immunity.

[60] Mohankumar V., Dhanushkodi N.R., Raju R. (2011). Sindbis virus replication, is insensitive to rapamycin and torin1, and suppresses Akt/mTOR pathway late during infection in HEK cells. Biochem. Biophys. Res. Commun..

[61] Patel R.K., Hardy R.W. (2012). Role for the phosphatidylinositol 3-kinase-Akt-TOR pathway during sindbis virus replication in arthropods. J. Virol..

[62] Shives K.D., Beatman E.L., Chamanian M., O’Brien C., Hobson-Peters J., Beckham J.D. (2014). West nile virus-induced activation of mammalian target of rapamycin complex 1 supports viral growth and viral protein expression. J. Virol..

[63] Urbanowski M.D., Hobman T.C. (2013). The West Nile virus capsid protein blocks apoptosis through a phosphatidylinositol 3-kinase-dependent mechanism. J. Virol..

[64] Lee C.J., Liao C.L., Lin Y.L. (2005). Flavivirus activates phosphatidylinositol 3-kinase signaling to block caspase-dependent apoptotic cell death at the early stage of virus infection. J. Virol..

[65] Gavrilovskaya I.N., Gorbunova E.E., Mackow E.R. (2013). Hypoxia induces permeability and giant cell responses of Andes virus-infected pulmonary endothelial cells by activating the mTOR-S6K signaling pathway. J. Virol..

[66] McNulty S., Flint M., Nichol S.T., Spiropoulou C.F. (2013). Host mTORC1 signaling regulates andes virus replication. J. Virol..

[67] Hale B.G., Jackson D., Chen Y.H., Lamb R.A., Randall R.E. (2006). Influenza A virus NS1 protein binds p85beta and activates phosphatidylinositol-3-kinase signaling. Proc. Natl. Acad. Sci. USA.

[68] Shin Y.K., Liu Q., Tikoo S.K., Babiuk L.A., Zhou Y. (2007). Effect of the phosphatidylinositol 3-kinase/Akt pathway on influenza A virus propagation. J. Gen. Virol..

[69] Avota E., Avots A., Niewiesk S., Kane L.P., Bommhardt U., ter Meulen V., Schneider-Schaulies S. (2001). Disruption of Akt kinase activation is important for immunosuppression induced by measles virus. Nat. Med..

[70] de Souza A.P., de Freitas D.N., Antuntes Fernandes K.E., D’Avila da Cunha M., Antunes Fernandes J.L., Benetti Gassen R., Fazolo T., Pinto L.A., Scotta M., Mattiello R. (2016). Respiratory syncytial virus induces phosphorylation of mTOR at ser2448 in CD8 T cells from nasal washes of infected infants. Clin. Exp. Immunol..

[71] Moy R.H., Gold B., Molleston J.M., Schad V., Yanger K., Salzano M.V., Yagi Y., Fitzgerald K.A., Stanger B.Z., Soldan S.S. (2014). Antiviral autophagy restrictsRift Valley fever virus infection and is conserved from flies to mammals. Immunity.

[72] Dunn E.F., Connor J.H. (2011). Dominant inhibition of Akt/protein kinase B signaling by the matrix protein of a negative-strand RNA virus. J. Virol..

[73] Cantley L.C., Neel B.G. (1999). New insights into tumor suppression: PTEN suppresses tumor formation by restraining the phosphoinositide 3-kinase/AKT pathway. Proc. Natl. Acad. Sci. USA.

[74] Cully M., You H., Levine A.J., Mak T.W. (2006). Beyond PTEN mutations: The PI3K pathway as an integrator of multiple inputs during tumorigenesis. Nat. Rev. Cancer.

[75] Hemmings B.A., Restuccia D.F. (2012). PI3K-PKB/Akt pathway. Cold Spring Harb. Perspect. Biol..

[76] Kong D., Yamori T. (2008). Phosphatidylinositol 3-kinase inhibitors: Promising drug candidates for cancer therapy. Cancer Sci..

[77] O’Shea C.C., Choi S., McCormick F., Stokoe D. (2005). Adenovirus overrides cellular checkpoints for protein translation. Cell Cycle.

[78] Sheng M., Sala C. (2001). PDZ domains and the organization of supramolecular complexes. Annu. Rev. Neurosci..

[79] Frese K.K., Lee S.S., Thomas D.L., Latorre I.J., Weiss R.S., Glaunsinger B.A., Javier R.T. (2003). Selective PDZ protein-dependent stimulation of phosphatidylinositol 3-kinase by the adenovirus E4-ORF1 oncoprotein. Oncogene.

[80] Scholle F., Bendt K.M., Raab-Traub N. (2000). Epstein-Barr virus LMP2A transforms epithelial cells, inhibits cell differentiation, and activates Akt. J. Virol..

[81] Lin Z., Wan X., Jiang R., Deng L., Gao Y., Tang J., Yang Y., Zhao W., Yan X., Yao K. (2014). Epstein-Barr virus-encoded latent membrane protein 2A promotes the epithelial-mesenchymal transition in nasopharyngeal carcinoma via metastatic tumor antigen 1 and mechanistic target of rapamycin signaling induction. J. Virol..

[82] Toh Y., Pencil S.D., Nicolson G.L. (1994). A novel candidate metastasis-associated gene, mta1, differentially expressed in highly metastatic mammary adenocarcinoma cell lines. cDNA cloning, expression, and protein analyses. J. Biol. Chem..

[83] Martin D., Nguyen Q., Molinolo A., Gutkind J.S. (2014). Accumulation of dephosphorylated 4EBP after mTOR inhibition with rapamycin is sufficient to disrupt paracrine transformation by the KSHV vGPCR oncogene. Oncogene.

[84] Stallone G., Schena A., Infante B., Di Paolo S., Loverre A., Maggio G., Ranieri E., Gesualdo L., Schena F.P., Grandaliano G. (2005). Sirolimus for Kaposi’s sarcoma in renal-transplant recipients. N. Engl. J. Med..

[85] Campistol J.M., Gutierrez-Dalmau A., Torregrosa J.V. (2004). Conversion to sirolimus: a successful treatment for posttransplantation Kaposi’s sarcoma. Transplantation.

[86] Beatman E., Oyer R., Shives K.D., Hedman K., Brault A.C., Tyler K.L., Beckham J.D. (2012). West Nile virus growth is independent of autophagy activation. Virology.

[87] Gottlieb K.A., Villarreal L.P. (2001). Natural biology of polyomavirus middle T antigen. Microbiol. Mol. Biol. Rev..

[88] Yuan H., Veldman T., Rundell K., Schlegel R. (2002). Simian virus 40 small tumor antigen activates AKT and telomerase and induces anchorage-independent growth of human epithelial cells. J. Virol..

[89] Spuul P., Balistreri G., Kaariainen L., Ahola T. (2010). Phosphatidylinositol 3-kinase-, actin-, and microtubule-dependent transport of Semliki Forest Virus replication complexes from the plasma membrane to modified lysosomes. J. Virol..

[90] Abere B., Wikan N., Ubol S., Auewarakul P., Paemanee A., Kittisenachai S., Roytrakul S., Smith D.R. (2012). Proteomic analysis of chikungunya virus infected microgial cells. PLoS ONE.

[91] Bianchini A., Loiarro M., Bielli P., Busa R., Paronetto M.P., Loreni F., Geremia R., Sette C. (2008). Phosphorylation of eIF4E by MNKs supports protein synthesis, cell cycle progression and proliferation in prostate cancer cells. Carcinogenesis.

[92] Fesq H., Bacher M., Nain M., Gemsa D. (1994). Programmed cell death (apoptosis) in human monocytes infected by influenza A virus. Immunobiology.

[93] Hinshaw V.S., Olsen C.W., Dybdahl-Sissoko N., Evans D. (1994). Apoptosis: A mechanism of cell killing by influenza A and B viruses. J. Virol..

[94] Ehrhardt C., Marjuki H., Wolff T., Nurnberg B., Planz O., Pleschka S., Ludwig S. (2006). Bivalent role of the phosphatidylinositol-3-kinase (PI3K) during influenza virus infection and host cell defence. Cell Microbiol..

[95] Zhou Z., Jiang X., Liu D., Fan Z., Hu X., Yan J., Wang M., Gao G.F. (2009). Autophagy is involved in influenza A virus replication. Autophagy.

[96] Datan E., Shirazian A., Benjamin S., Matassov D., Tinari A., Malorni W., Lockshin R.A., Garcia-Sastre A., Zakeri Z. (2014). mTOR/p70S6K signaling distinguishes routine, maintenance-level autophagy from autophagic cell death during influenza A infection. Virology.

[97] Zhirnov O.P., Konakova T.E., Garten W., Klenk H. (1999). Caspase-dependent N-terminal cleavage of influenza virus nucleocapsid protein in infected cells. J. Virol..

[98] Liu G., Zhong M., Guo C., Komatsu M., Xu J., Wang Y., Kitazato K. (2016). Autophagy is involved in regulating influenza A virus RNA and protein synthesis associated with both modulation of Hsp90 induction and mTOR/p70S6K signaling pathway. Int. J. Biochem. Cell Biol..

[99] Gannage M., Dormann D., Albrecht R., Dengjel J., Torossi T., Ramer P.C., Lee M., Strowig T., Arrey F., Conenello G. (2009). Matrix protein 2 of influenza A virus blocks autophagosome fusion with lysosomes. Cell Host Microbe.

[100] Soares J.A., Leite F.G., Andrade L.G., Torres A.A., De Sousa L.P., Barcelos L.S., Teixeira M.M., Ferreira P.C., Kroon E.G., Souto-Padron T. (2009). Activation of the PI3K/Akt pathway early during vaccinia and cowpox virus infections is required for both host survival and viral replication. J. Virol..

[101] Allan G.M., McNeilly F., Cassidy J.P., Reilly G.A., Adair B., Ellis W.A., McNulty M.S. (1995). Pathogenesis of porcine circovirus; experimental infections of colostrum deprived piglets and examination of pig foetal material. Vet. Microbiol..

[102] Zhu B., Xu F., Li J., Shuai J., Li X., Fang W. (2012). Porcine circovirus type 2 explores the autophagic machinery for replication in PK-15 cells. Virus Res..

[103] Zhu B., Zhou Y., Xu F., Shuai J., Li X., Fang W. (2012). Porcine circovirus type 2 induces autophagy via the AMPK/ERK/TSC2/mTOR signaling pathway in PK-15 cells. J. Virol..

[104] Manning B.D., Cantley L.C. (2007). AKT/PKB signaling: Navigating downstream. Cell.

[105] Clippinger A.J., Maguire T.G., Alwine J.C. (2011). Human cytomegalovirus infection maintains mTOR activity and its perinuclear localization during amino acid deprivation. J. Virol..

[106] Kudchodkar S.B., Del Prete G.Q., Maguire T.G., Alwine J.C. (2007). AMPK-mediated inhibition of mTOR kinase is circumvented during immediate-early times of human cytomegalovirus infection. J. Virol..

[107] Tilton C., Clippinger A.J., Maguire T., Alwine J.C. (2011). Human cytomegalovirus induces multiple means to combat reactive oxygen species. J. Virol..

[108] Werden S.J., McFadden G. (2010). Pharmacological manipulation of the akt signaling pathway regulates myxoma virus replication and tropism in human cancer cells. J. Virol..

[109] Stanford M.M., Barrett J.W., Nazarian S.H., Werden S., McFadden G. (2007). Oncolytic virotherapy synergism with signaling inhibitors: Rapamycin increases myxoma virus tropism for human tumor cells. J. Virol..

[110] Connor J.H., Lyles D.S. (2002). Vesicular stomatitis virus infection alters the eIF4F translation initiation complex and causes dephosphorylation of the eIF4E binding protein 4E-BP1. J. Virol..

[111] Hopkins K.C., Tartell M.A., Herrmann C., Hackett B.A., Taschuk F., Panda D., Menghani S.V., Sabin L.R., Cherry S. (2015). Virus-induced translational arrest through 4EBP1/2-dependent decay of 5'-TOP mRNAs restricts viral infection. Proc. Natl. Acad. Sci. USA.

[112] Carsillo M., Kim D., Niewiesk S. (2010). Role of AKT kinase in measles virus replication. J. Virol..

[113] Bueno S.M., Gonzalez P.A., Pacheco R., Leiva E.D., Cautivo K.M., Tobar H.E., Mora J.E., Prado C.E., Zuniga J.P., Jimenez J. (2008). Host immunity during RSV pathogenesis. Int. Immunopharmacol..

[114] Araki K., Turner A.P., Shaffer V.O., Gangappa S., Keller S.A., Bachmann M.F., Larsen C.P., Ahmed R. (2009). mTOR regulates memory CD8 T-cell differentiation. Nature.

[115] Murray J.L., McDonald N.J., Sheng J., Shaw M.W., Hodge T.W., Rubin D.H., O’Brien W.A., Smee D.F. (2012). Inhibition of influenza A virus replication by antagonism of a PI3K-AKT-mTOR pathway member identified by gene-trap insertional mutagenesis. Antivir. Chem. Chemother..

[116] Zhang Y., Gao X., Saucedo L.J., Ru B., Edgar B.A., Pan D. (2003). Rheb is a direct target of the tuberous sclerosis tumour suppressor proteins. Nat. Cell Biol..

[117] Zhang L., Wu J., Ling M.T., Zhao L., Zhao K.N. (2015). The role of the PI3K/Akt/mTOR signalling pathway in human cancers induced by infection with human papillomaviruses. Mol. Cancer.

[118] Spangle J.M., Munger K. (2010). The human papillomavirus type 16 E6 oncoprotein activates mTORC1 signaling and increases protein synthesis. J. Virol..

[119] Terhune S., Torigoi E., Moorman N., Silva M., Qian Z., Shenk T., Yu D. (2007). Human cytomegalovirus UL38 protein blocks apoptosis. J. Virol..

[120] Xuan B., Qian Z., Torigoi E., Yu D. (2009). Human cytomegalovirus protein pUL38 induces ATF4 expression, inhibits persistent JNK phosphorylation, and suppresses endoplasmic reticulum stress-induced cell death. J. Virol..

[121] Bai Y., Xuan B., Liu H., Zhong J., Yu D., Qian Z. (2015). Tuberous Sclerosis Complex Protein 2-Independent Activation of mTORC1 by Human Cytomegalovirus pUL38. J. Virol..

[122] Rauwel B., Jang S.M., Cassano M., Kapopoulou A., Barde I., Trono D. (2015). Release of human cytomegalovirus from latency by a KAP1/TRIM28 phosphorylation switch. Elife.

[123] Benetti L., Roizman B. (2006). Protein kinase B/Akt is present in activated form throughout the entire replicative cycle of deltaU(S)3 mutant virus but only at early times after infection with wild-type herpes simplex virus 1. J. Virol..

[124] Chuluunbaatar U., Mohr I. (2011). A herpesvirus kinase that masquerades as Akt: You don’t have to look like Akt, to act like it. Cell Cycle.

[125] Gingras A.C., Kennedy S.G., O’Leary M.A., Sonenberg N., Hay N. (1998). 4E-BP1, a repressor of mRNA translation, is phosphorylated and inactivated by the Akt(PKB) signaling pathway. Genes Dev..

[126] Montero H., Garcia-Roman R., Mora S.I. (2015). eIF4E as a control target for viruses. Viruses.

[127] Kudchodkar S.B., Yu Y., Maguire T.G., Alwine J.C. (2004). Human cytomegalovirus infection induces rapamycin-insensitive phosphorylation of downstream effectors of mTOR kinase. J. Virol..

[128] Roizman B., Knipe D. (2001). Herpes Simplex Viruses and Their Replication.

[129] Feng H., Shuda M., Chang Y., Moore P.S. (2008). Clonal integration of a polyomavirus in human Merkel cell carcinoma. Science.

[130] Shuda M., Chang Y., Moore P.S. (2014). Merkel cell polyomavirus-positive Merkel cell carcinoma requires viral small T-antigen for cell proliferation. J. Investig. Dermatol..

[131] Shuda M., Velasquez C., Cheng E., Cordek D.G., Kwun H.J., Chang Y., Moore P.S. (2015). CDK1 substitutes for mTOR kinase to activate mitotic cap-dependent protein translation. Proc. Natl. Acad. Sci. USA.

[132] Yu Y., Kudchodkar S.B., Alwine J.C. (2005). Effects of simian virus 40 large and small tumor antigens on mammalian target of rapamycin signaling: Small tumor antigen mediates hypophosphorylation of eIF4E-binding protein 1 late in infection. J. Virol..

[133] Yu Y., Alwine J.C. (2006). 19S late mRNAs of simian virus 40 have an internal ribosome entry site upstream of the virion structural protein 3 coding sequence. J. Virol..

[134] Mannova P., Beretta L. (2005). Activation of the N-Ras-PI3K-Akt-mTOR pathway by hepatitis C virus: Control of cell survival and viral replication. J. Virol..

[135] Panda S., Vedagiri D., Viveka T.S., Harshan K.H. (2014). A unique phosphorylation-dependent eIF4E assembly on 40S ribosomes co-ordinated by hepatitis C virus protein NS5A that activates internal ribosome entry site translation. Biochem. J..

[136] Huang H., Kang R., Wang J., Luo G., Yang W., Zhao Z. (2013). Hepatitis C virus inhibits AKT-tuberous sclerosis complex (TSC), the mechanistic target of rapamycin (MTOR) pathway, through endoplasmic reticulum stress to induce autophagy. Autophagy.

[137] Shrivastava S., Bhanja Chowdhury J., Steele R., Ray R., Ray R.B. (2012). Hepatitis C virus upregulates Beclin1 for induction of autophagy and activates mTOR signaling. J. Virol..

[138] Dreux M., Chisari F.V. (2009). Autophagy proteins promote hepatitis C virus replication. Autophagy.

[139] Dreux M., Gastaminza P., Wieland S.F., Chisari F.V. (2009). The autophagy machinery is required to initiate hepatitis C virus replication. Proc. Natl. Acad. Sci. USA.

[140] Hamel R., Dejarnac O., Wichit S., Ekchariyawat P., Neyret A., Luplertlop N., Perera-Lecoin M., Surasombatpattana P., Talignani L., Thomas F. (2015). Biology of Zika Virus Infection in Human Skin Cells. J. Virol..

[141] Vandergaast R., Fredericksen B.L. (2012). West Nile virus (WNV) replication is independent of autophagy in mammalian cells. PLoS ONE.

[142] Kim E., Goraksha-Hicks P., Li L., Neufeld T.P., Guan K.L. (2008). Regulation of TORC1 by Rag GTPases in nutrient response. Nat. Cell Biol..

[143] Sancak Y., Peterson T.R., Shaul Y.D., Lindquist R.A., Thoreen C.C., Bar-Peled L., Sabatini D.M. (2008). The Rag GTPases bind raptor and mediate amino acid signaling to mTORC1. Science.

[144] Castillo C., Naranjo J., Sepulveda A., Ossa G., Levy H. (2001). Hantavirus pulmonary syndrome due to Andes virus in Temuco, Chile: Clinical experience with 16 adults. Chest.

[145] Lopez N., Padula P., Rossi C., Lazaro M.E., Franze-Fernandez M.T. (1996). Genetic identification of a new hantavirus causing severe pulmonary syndrome in Argentina. Virology.

[146] Daussy C.F., Beaumelle B., Espert L. (2015). Autophagy restricts HIV-1 infection. Oncotarget.

[147] Dinkins C., Pilli M., Kehrl J.H. (2015). Roles of autophagy in HIV infection. Immunol. Cell Biol..

[148] Campbell G.R., Rawat P., Bruckman R.S., Spector S.A. (2015). Human Immunodeficiency Virus Type 1 Nef Inhibits Autophagy through Transcription Factor EB Sequestration. PLoS Pathog..

[149] Cinti A., Le Sage V., Milev M.P., Crossie C., Valiente-Echeverría F., Olivier F., Mouland A.J. HIV-1 enhances mTORC1 activity and repositions lysosomes to the periphery by co-opting Rag GTPases.

[150] Nardacci R., Amendola A., Ciccosanti F., Corazzari M., Esposito V., Vlassi C., Taibi C., Fimia G.M., Del Nonno F., Ippolito G. (2014). Autophagy plays an important role in the containment of HIV-1 in nonprogressor-infected patients. Autophagy.

[151] Donia M., McCubrey J.A., Bendtzen K., Nicoletti F. (2010). Potential use of rapamycin in HIV infection. Br. J. Clin. Pharmacol..

[152] Heredia A., Le N., Gartenhaus R.B., Sausville E., Medina-Moreno S., Zapata J.C., Davis C., Gallo R.C., Redfield R.R. (2015). Targeting of mTOR catalytic site inhibits multiple steps of the HIV-1 lifecycle and suppresses HIV-1 viremia in humanized mice. Proc. Natl. Acad. Sci. USA.

